# Functional Characterization of a Small Alarmone Hydrolase in *Corynebacterium glutamicum*

**DOI:** 10.3389/fmicb.2018.00916

**Published:** 2018-05-09

**Authors:** Matthias Ruwe, Christian Rückert, Jörn Kalinowski, Marcus Persicke

**Affiliations:** Microbial Genomics and Biotechnology, Center for Biotechnology, Bielefeld University, Bielefeld, Germany

**Keywords:** stringent response, alarmone, (p)ppGpp, (pp)pGpp, Mesh1-L, RelH, SAH, pGpp

## Abstract

The (pp)pGpp metabolism is an important component of bacterial physiology as it is involved in various stress responses and mechanisms of cell homeostasis, e.g., the regulation of growth. However, in order to better understand the (pp)pGpp associated regulation, it is crucial to study the molecular mechanisms of (pp)pGpp metabolism. In recent years, bioinformatic analyses of the RelA/SpoT homolog (RSH) superfamily have led to the discovery of small monofunctional RSH derivatives in addition to the well-known bifunctional Rel proteins. These are also referred to as small alarmone synthetases (SASs) or small alarmone hydrolases (SAHs). In this study, the ORF *cg1485* from *C. glutamicum* was identified as a putative SAH encoding gene, based on a high similarity of the corresponding amino acid sequence with the (pp)pGpp hydrolysis domain. The characterization of its gene product, designated as RelH_Cg_, represents the first functional investigation of a bacterial representative of the SAH subfamily. The predicted pyrophosphohydrolase activity was demonstrated *in vivo* by expression in two *E. coli* strains, characterized by different alarmone basal levels, as well as by *in vitro* analysis of the purified protein. During the assay-based analysis of hydrolysis activity in relation to the three known alarmone species, both RelH_Cg_ and the bifunctional RSH enzyme Rel_Cg_ were found to exhibit a pronounced substrate inhibition for alarmone concentrations of more than 0.75 mM. This characteristic of (pp)pGpp hydrolases could be an important mechanism for realizing the bistable character of the (pp)pGpp metabolism between a (pp)pGpp basal level and stress-associated alarmone production. The deletion of *relH*_Cg_ caused only a minor effect on growth behavior in both wild-type background and deletion mutants with deletion of (pp)pGpp synthetases. Based on this observation, the protein is probably only present or active under specific environmental conditions. The independent loss of the corresponding gene in numerous representatives of the genus *Corynebacterium*, which was found by bioinformatic analyses, also supports this hypothesis. Furthermore, growth analysis of all possible deletion combinations of the three active *C. glutamicum* RSH genes revealed interesting functional relationships which will have to be investigated in more detail in the future.

## Introduction

The stringent response represents an important bacterial regulatory system, as it allows the organisms to respond quickly to different stress conditions. In response to unfavorable environmental conditions such as nutrient deficiency situations, the hyperphosphorylated guanosine derivatives GMP 3′diphosphate (pGpp) (Gaca et al., 2015b), guanosine tetraphosphate (ppGpp) and guanosine pentaphosphate (pppGpp) are produced (Cashel and Kalbacher, 1970; Haseltine et al., 1972). These messenger nucleotides, also referred to as alarmones or as (pp)pGpp, influence the expression or activity of numerous proteins and thereby orchestrate the inhibition of cell growth as well as the redistribution of cellular resources toward a state of persistence (Dalebroux and Swanson, 2012; Gaca et al., 2015a). New results also illustrate that the (pp)pGpp metabolism, which is highly conserved in bacteria and plant chloroplasts (van der Biezen et al., 2000; Masuda et al., 2008; Potrykus and Cashel, 2008), not only induces the coordinated response to various stress conditions, but also includes numerous regulatory functions for the maintenance of cell homeostasis (Potrykus and Cashel, 2008; Gaca et al., 2013) and growth rate regulation (Potrykus et al., 2011). Due to their functions in the context of stress-induced (pp)pGpp production, alarmone synthetases have long been in the focus of research. However, especially when considering the non-stress-associated functions of (pp)pGpp metabolism, the counteracting (pp)pGpp hydrolysis is likewise an important and so far little studied component of this system (Hauryliuk et al., 2015). The importance of (pp)pGpp hydrolases has already been demonstrated by the fact that the deletion of the only gene with this function in the model organism *Escherichia coli*, termed *spoT*_Ec_, is lethal (Xiao et al., 1991). Due to the wide-ranging regulatory influences of the (pp)pGpp metabolism, a deeper understanding of this system is highly relevant for medical areas such as stress associated persistence of pathogenic species (Dalebroux et al., 2010), as well as for biotechnological applications (Michalowski et al., 2017).

The enzymes responsible for synthesis and hydrolysis of (pp)pGpp are referred to as RelA/SpoT homologs (RSHs) because of their protein sequence similarities to the corresponding *E. coli* proteins RelA_Ec_ and SpoT_Ec_. However, in most organisms only one copy of this multi-domain enzyme occurs, which also contains other domains associated with regulatory functions. While the pyrophosphokinase activity has already been extensively studied due to its high relevance in the context of various stress responses, the pyrophosphohydrolase activity mediating HD domain of RSH proteins has so far been functionally characterized only for a few organisms (Aravind and Koonin, 1998). In addition to the ‘long’ multi-domain RSH enzymes which have significantly divergent activity spectra due to different regulatory mechanisms, small single-domain RSH proteins have recently been identified by bioinformatic approaches (Lemos et al., 2007; Nanamiya et al., 2008; Sun et al., 2010). These include monofunctional (pp)pGpp synthetases as well as (pp)pGpp hydrolases, also referred to as small alarmone synthetases (SASs) and small alarmone hydrolases (SAHs), respectively. A bioinformatics study of the RSH superfamily carried out by Atkinson et al. (2011) revealed the widespread occurrence of these two protein classes. By searching for stringent response-associated sequence motifs in 1,000 genomes and a subsequent phylogenetic analysis of the identified genes, a classification into 12 subgroups of SAS proteins and 7 subgroups of SAH enzymes has been proposed.

In addition to the bifunctional enzyme Rel_Cg_, the presence of the SAS enzyme RelS_Cg_ was recently reported for the industrially relevant production organism *C. glutamicum* (Ruwe et al., 2017). The deletion of the *C. glutamicum rel*_Cg_ gene resulted in a growth deficit in minimal medium, probably caused by increased (pp)pGpp levels due to the synthetase activity of RelS_Cg_ and a lacking hydrolase activity by Rel_Cg_. Contrary to expectations, however, after a stationary phase lasting several hours in the low OD range, the strain began to grow again with almost normal parameters. Based on these observations the existence of a further (pp)pGpp hydrolase was assumed. A candidate protein has already been identified by bioinformatic analysis of putative *C. glutamicum* SAS enzymes (Ruwe et al., 2017), as well as by Atkinson et al. (2011) in the context of a global RSH classification based on amino acid sequence similarities. However, regarding this class of enzymes nearly no information is available. The only members of the SAH proteins investigated so far are eukaryotic representatives of *Drosophila melanogaster* and *Homo sapiens* (Sun et al., 2010). However, as no (pp)pGpp synthetases are known for both organisms, the functional context of these proteins is completely unknown and may deviate from those of the prokaryotic orthologs.

In addition, the production of pGpp was found for both (pp)pGpp synthetases in *C. glutamicum* in the course of our previous investigations. The assembly and disassembly of this substance, which has only recently been assigned to the alarmone species, could be an important link within the stringent response of *C. glutamicum* and related organisms. Since different biological functionalities have been identified for the three known alarmone species in recent years (Gaca et al., 2015b), differences in the activity of the hydrolytically active enzymes with regard to these substrates could represent an important and so far unexplored component of the (pp)pGpp metabolism.

The aim of the work described here is the analysis of a possible expansion of the *C. glutamicum* (pp)pGpp metabolism by enzymes with (pp)pGpp hydrolase activity and a further elucidation of the functional relationship between the different RSH species. By heterologous expression of a *C. glutamicum* SAH gene in *E. coli* and the development of a LC-MS based, *in vitro* (pp)pGpp hydrolase characterization method, we were able to confirm the predicted functionality and for the first time provide enzyme kinetics data for a bacterial monofunctional small alarmone hydrolase. Both, for the pyrophosphohydrolase activities of Rel_Cg_ and the newly identified SAH enzyme, a significant substrate inhibition was found, which could be an important mechanistic component for realizing the bistable character of (pp)pGpp metabolism.

## Materials and Methods

### Bacterial Strains and Growth Conditions

Bacterial strains used or constructed for this study are listed in **Table 1**, and plasmids are listed in **Table 2**.

**Table 1:** Bacterial strains used in this study.

Strain	Characteristics or genotype	Source
*C. glutamicum*		
CR099	*C. glutamicum* ATCC 13032, ΔCGP1, ΔCGP2, ΔCGP3, ΔISCg1, ΔISCg2	Baumgart et al., 2013; Unthan et al., 2015
CR099 *Δrel*	*C. glutamicum* CR099 Δ*rel*	Ruwe et al., 2017
CR099 Δ*relS*	*C. glutamicum* CR099 Δ*relS*	Ruwe et al., 2017
CR099 Δ*relH*	*C. glutamicum* CR099 Δ*relH*	This study
CR099 Δ*rel*Δ*relS*	*C. glutamicum* CR099 Δ*rel*Δ*relS*	Ruwe et al., 2017
CR099 Δ*rel*Δ*relH*	*C. glutamicum* CR099 Δ*rel*Δ*relH*	This study
CR099 Δ*relS*Δ*relH*	*C. glutamicum* CR099 Δ*relS*Δ*relH*	This study
CR099 Δ*rel*Δ*relS*Δ*relH*	*C. glutamicum* CR099 Δ*rel*Δ*relS*Δ*relH*	This study
*E. coli*		
ER2566	*E. coli* expression strain with a chromosomal copy of the T7 RNA polymerase gene under the control of the lac promoter	New England Biolabs
MG1655	Wild-type *E. coli* MG1655, derived from *E. coli* K12	Blattner et al., 1997
MG1655 Δ*relA*	MG1655 *ΔrelA*	Ruwe et al., 2017

**Table 2:** Plasmids used in this study.

Plasmid	Characteristics	Source
pZMP	Constitutive *E. coli* – *C. glutamicum* shuttle expression vector	Walter et al., 2016
pZMP::*rel*_Cg_	pZMP carrying the gene *rel* (*cg1861*) from *C. glutamicum* CR099	This study
pZMP::*relS*_Cg_	pZMP carrying the gene *relS* (*cg2324*) from *C. glutamicum* CR099	This study
pZMP::*relH*_Cg_	pZMP carrying the gene *relH* (*cg1485*) from *C. glutamicum* CR099	This study
pK18*mobsacB*	Suicide vector for gene deletion by homologous recombination	Schäfer et al., 1994
pK18*mobsacB*_*relH*_Cg_	pK18*mobsacB* carrying the up- and downstream region (500 bp, respectively) of the gene *relH* from *C. glutamicum* CR099	This study
pTXB1	*E. coli* expression vector, carrying the self-cleavable *Mxe* intein/chitin binding domain	New England Biolabs
pTXB1::*rel*_Cg_	pTXB1 carrying the gene *rel* (*cg1861*) from *C. glutamicum* CR099	Ruwe et al., 2017
pTXB1::*relS*_Cg_	pTXB1 carrying the gene *relS* (*cg2324*) from *C. glutamicum* CR099	Ruwe et al., 2017
pTXB1::*relH*_Cg_	pTXB1 carrying the gene *relH* (*cg1485*) from *C. glutamicum* CR099	This study

*C. glutamicum* and *E. coli* strains were cultivated as described previously (Ruwe et al., 2017). In order to avoid the accumulation of pseudorevertants in the precultures of the minimal medium cultivation, all strains were grown in CASO broth (Carl Roth) and subsequently washed in the chemically defined CGXII main culture medium (Keilhauer et al., 1993).

### Construction of *C. glutamicum* Deletion Mutants

The strains used in this study were constructed as described previously (Ruwe et al., 2017). All primers used in this study are listed in Supplementary Table S1 with their respective sequences and purposes. Briefly, *C. glutamicum* deletion mutants were generated using the pK18*mobsacB* suicide vector system (Schäfer et al., 1994).

### Constitutive Expression of RSH Genes in *E. coli* MG1655 and *E. coli* MG1655 Δ*relA*

In order to investigate the *in vivo* activity of putative (pp)pGpp pyrophosphohydrolases from *C. glutamicum*, a constitutive expression of the corresponding genes in two *E. coli* test strains with different (pp)pGpp basal levels was aimed at. For this purpose, the genes *cg1861* (*rel*_Cg_), *cg1485* (*relH*_Cg_) and the SAS gene *cg2324* (*relS*_Cg_) were cloned into the vector pZMP by means of Gibson Isothermal Assembly under control of the *tac* promoter (Gibson et al., 2009). The resulting plasmids pZMP::*rel*_Cg_, pZMP::*relH*_Cg_ and pZMP::*relS*_Cg_, as well as the empty plasmid were subsequently transformed into the strains *E. coli* MG1655 and *E. coli* MG1655 Δ*relA*.

All strains were cultivated in LB medium up to an OD_600_ of 3, washed in PBS buffer (pH 7.4) and adjusted to an OD_600_ of 0.1. Subsequently, 5 μL aliquots of different dilution stages were spotted onto LB-medium plates, MOPS minimal medium plates (MM) and MOPS minimal medium plates containing each 1 mM L-serine, L-methionine, and L-glycine (MM+SMG) (Kasai, 2002). All solid media plates contained 50 μg mL^-1^ kanamycin and 4 g L^-1^ glycerol was used as C source for all minimal medium plates. Incubation was carried out at 37°C for 24 h in the case of LB solid medium and 48 h for MM and MM+SMG plates.

### Protein Purification

For the tag-free purification of the putative SAH enzyme RelH_Cg_, the IMPACT kit (New England Biolabs) was used analogously to the purification of the already available enzymes Rel_Cg_ and RelS_Cg_, which were also used in the course of this study. The corresponding gene *relH*_Cg_ was introduced into the vector pTXB1 and the plasmid obtained was transformed into the expression strain *E. coli* ER2566. The further procedure was consistent with the purification of Rel_Cg_ and RelS_Cg_ already described in the previous study (Ruwe et al., 2017).

### Preparation of pGpp, ppGpp, and pppGpp

The three alarmone species pGpp, ppGpp, and pppGpp were produced by RelS_Cg_-catalyzed pyrophosphorylation of GMP, GDP or GTP and purified by chromatography. The procedure was based on a protocol for the production and purification of ppGpp and pppGpp by Mechold et al. (2013). However, it was adapted to the usage of RelS_Cg_, optimized in terms of optimum purity, and modified for the purification of pGpp. The reaction mixtures comprising 2.5 mL contained 50 mM Tris-HCl (pH 8), 10 mM MgCl_2_, 12 mM ATP, 10 mM of the respective guanosine derivative and 1.5 μM purified RelS_Cg_. After an incubation period of 16 h, phenol chloroform extraction was performed, using an equivalent volume of phenol chloroform isoamyl alcohol mixture (25:24:1). Nucleotides were precipitated by adjusting the LiCl concentration to 1 M and adding four volumes of ice-cold absolute ethanol. Following an incubation period of 30 min on ice, the suspension was centrifuged at 4°C and 7,200 × *g* for 10 min. The obtained pellet was washed two times with absolute ethanol, dried on ice and stored at -20°C.

For purification, the precipitated guanosine derivatives were resuspended in binding buffer [0.5 mM EDTA, 25 mM TRIS-HCl (pH 7.5)], containing 50 mM LiCl and loaded onto a 5 mL Bio-Scale Mini Macro-Prep High Q Cartridge (Bio-Rad), using an ÄKTAprime plus system. Following a washing step at a LiCl concentration of 150 mM, the elution of ppGpp and pppGpp was carried out with a linear gradient of 150–500 mM LiCl in binding buffer. In the case of pGpp, the LiCl concentration was increased in two sections with different gradient slopes from 150 mM to 350 mM and 350 to 500 mM to achieve optimal separation. The eluate was collected in 2 mL fractions. In order to allocate the obtained UV signals to the nucleotide components contained, representative fractions were investigated by means of HPLC-MS measurements using a pppGpp pyrophosphokinase assay HPLC-MS method as described previously (Ruwe et al., 2017). The fractions corresponding to the desired guanosine derivatives were combined, diluted with LiCl-free binding buffer in a ratio of 1:6 and loaded again onto the column mentioned above. This was followed by a batch elution with binding buffer containing 1 M LiCl and a precipitation as described above. The concentration of all alarmone species dissolved in water was determined by UV absorption measurement.

### (pp)pGpp Pyrophosphohydrolase Assay

In order to characterize (pp)pGpp hydrolysis activities of Rel_Cg_ and RelH_Cg_, the enzymes were incubated with different alarmone species under variation of relevant process parameters. The enzyme reactions, each containing 50 μL, were stopped by a strong reduction of the pH. For this purpose, 4 μL 50% acetic acid was added, which corresponds to an optimal ratio between preferably complete inactivation and the smallest possible decay of the substrates or products. HPLC analysis of the hydrolase assays was based on the previously described method for the detection of (pp)Gpp in pyrophosphokinase assays (Ruwe et al., 2017). The concentration of the isocratic elution profile was adapted to the analyzed nucleotide species. For the evaluation of pppGpp hydrolysis 38% of 10 mM ammonium bicarbonate buffer (pH 9.3) was used. In contrast, 36% and 34% of this buffer were applied to analyze assay mixtures containing ppGpp or pGpp, respectively. The quantitative evaluation of the products GMP, GDP, or GTP was performed using the UV signal and suitable nucleotide standards, which were also incubated under identical conditions and treated with acetic acid. The reaction mixtures for the characterization of the pyrophosphohydrolase reaction with regard to divalent ions contained 50 mM HEPES-Na (pH 8.0), 200 mM NaCl, 0.5 mM ppGpp as well as 52.3 nM RelH_Cg_ or 98.2 nM Rel_Cg_, respectively. In order to avoid oxidation of Mn^2+^, the Mn^2+^ containing buffer solutions were saturated with nitrogen before the addition of substrates and enzymes to remove dissolved oxygen. Since comparatively low activities were measured using different MgCl_2_ values, 10 times higher enzyme concentrations were used. The incubation time was 25 min for RelH_Cg_ and 45 min for Rel_Cg_ containing approaches. The pH dependence was basically determined with identical reaction components and test parameters, although different buffer systems and a defined MnCl_2_ concentration of 1 mM were used. Citrate, phosphate, HEPES-Na, Tris-HCL and ammonia buffer systems with a concentration of 50 mM each were applied to cover a broad pH spectrum. For the highest and lowest pH values, enzyme-free controls were carried out to verify the stability of the substrate ppGpp over the entire pH range.

For the kinetic analysis of the pyrophosphohydrolase activities of Rel_Cg_ or RelH_Cg_, assays with different concentrations of the three alarmone species were performed. Since maximum ppGpp concentrations of 4 mM were determined for *E. coli*, the substrate concentrations were varied from 0.1 to 4 mM. The reaction mixtures comprised 50 mM HEPES-Na (pH 8.0), 200 mM NaCl, 1 mM MnCl_2_ and 52.3 nM RelH_Cg_ or 98.2 nM Rel_Cg_, respectively. The conditions used were based on the characterization of ppGpp hydrolysis with respect to different reaction parameters and represent a compromise between high activity of both enzymes and physiological conditions. In order to ensure optimal evaluability, the incubation times of the different enzyme-substrate combinations were adapted to the values determined in preliminary tests and ranged from 20 to 45 min. The incubation temperature was 30°C.

Data evaluation was carried out using the program OriginPro 2018 (OriginLab). During the analysis of the enzyme kinetics with respect to different substrate concentrations, a pronounced substrate inhibition was observed. Since no converging fit of the experimental data could be realized using classical functions to describe the enzyme kinetics in the case of substrate inhibition, the kinetic parameters *K*_m_, *k*_cat_ and the corresponding inhibition constants could not be determined conclusively. For this reason, the kinetic parameters *K*_m_ and *k*_cat_ were determined by a linearized representation according to Eadie–Hofstee using the lowest evaluable substrate concentrations (Hofstee, 1959). Only two data points could be used for two data sets in this context, so that no error analysis was possible and the determined parameters merely represent an approximation.

### Bioinformatic Analyses

To assess the distribution of RelH_Cg_ within the genus *Corynebacterium*, an HMM of RelH_Cg_ orthologs was constructed using the program JACKHMMER (Finn et al., 2015). The amino acid sequence of Cg1485 was used as an input against the ”Reference proteomes” database with an initial significance *e*-value of 1e-25. After four iterations, a large increase of candidates was observed, so the third iteration was inspected. Based on the observed minimum, an e-value of 1e-37 was chosen which led to convergence after five iterations with a total of 446 sequences giving hits above the cutoff. The HMM resulting from these sequences was then used as input for HMMSEARCH to search for RelH_Cg_ in the available proteomes of *Corynebacterium* species strains. Hits with an *e*-value below 1e-37 were considered significant. The data were mapped on a phylogenetic tree of the *Corynebacterium* type strains, created on the basis of the respective 16S rDNA sequences according to the RDP database (Cole et al., 2014).

## Results

### The Genome of *C. glutamicum* Encodes a Putative Small Alarmone Hydrolase (SAH) Containing All Conserved (pp)pGpp Hydrolase Sequence Motifs

To identify possible monofunctional (pp)pGpp hydrolases for *C. glutamicum*, a BLASTP analysis of the already known bifunctional enzyme Rel_Cg_ was performed with a *C. glutamicum* ATCC 13032 reference genome (Kalinowski et al., 2003). In the course of this investigation, a high degree of similarity was found between the Rel_Cg_ hydrolase domain and the amino acid sequence of the gene product of *cg1485*. This result is consistent with a global ‘high throughput sensitive sequence searching’ of the RSH superfamily, where the corresponding ORF was also identified as a putative SAH (Atkinson et al., 2011). The analysis of a pooled *C. glutamicum* RNAseq data set, based on cultures from different cultivation- and stress conditions, revealed the full-length transcription of the ORF *cg1485* (Pfeifer-Sancar et al., 2013). Based on a data set of native 5′-ends of transcripts, also created in the context of this study, a clear -10 motif was identified, which indicates a σ^70^ associated transcription. Hence, an expression of the SAH gene *cg1485* can be assumed. However, due to the pooling of samples from different conditions for the RNAseq experiments, the exact expression conditions are not known.

**FIGURE 1:**
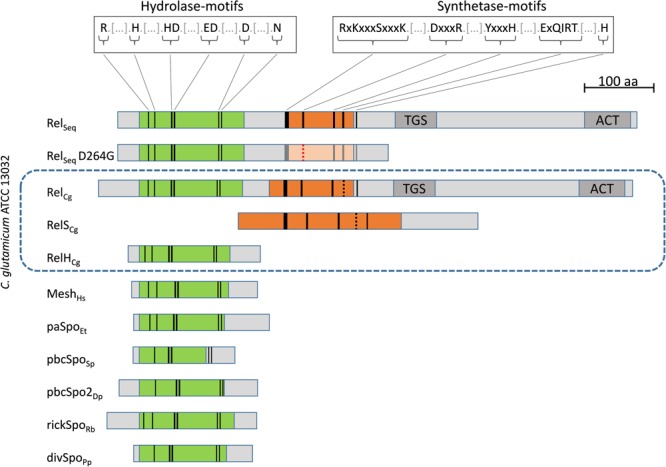
Comparative structural analysis of enzymes involved in (pp)pGpp metabolism. True-to-scale representation of domain architecture and conserved sequence motives of stringent response associated enzymes of *C. glutamicum* ATCC 13032 (enclosed by dashed blue lines) and further selected species. These include the native Rel enzyme of *Streptococcus equisimilis* Rel_Seq_, as well as the shortened and only hydrolytically active variant Rel_Seq_ D264G (Hogg et al., 2004; Sun et al., 2010) and representatives of the six other SAH subgroups (Atkinson et al., 2011): Mesh (*Homo sapiens*), paSpo (*Erwinia tasmaniensis*), pbcSpo (*S. pneumoniae*), pbcSpo2 (*Desulfotalea psychrophila*), rickSpo (*Rickettsia bellii*), and divSpo (*Photobacterium profundum*). Hydrolase domain HD4 (pfam13328) and ppGpp synthetase catalytic domain YjbM (COG2357) are indicated in green and orange, respectively. Additional C-terminal elements of long RSH enzymes, which are likely to have regulatory functions and are classified as TGS (ThrRS, GTPase, and SpoT) and ACT (aspartate kinase, chorismate mutase and TyrA) domains (Wolf et al., 1999; Chipman and Shaanan, 2001), are also indicated. Matches with conserved synthetase and hydrolase motifs are represented by black lines (Bag et al., 2014; Steinchen and Bange, 2016). Minor deviations from the conserved sequence motifs are shown by dashed black lines. The amino acid exchange of the protein Rel_Seq_ D264G is represented by a red dashed line and the resulting non-functional YjbM domain is faded out.

Based on a phylogenetic analysis of all SAH candidates found, Atkinson et al. (2011) proposed a differentiation of seven SAH subgroups and classified *cg1485* as a member of the Mesh1-L subgroup. However, since no suitable gene name has been assigned for representatives of this group, we here propose the term *relH* and subsequently designate the gene *cg1485* as *relH*_Cg_. The corresponding enzyme RelH_Cg_ is attributed to the metal-dependent phosphohydrolases due to its high level of similarity to the HD_4 domain (pfam13328) (Aravind and Koonin, 1998). In addition, the amino acid sequence of the *relH*_Cg_ gene product is consistent with all 6 conserved catalytic amino acid sequence motifs identified for the (pp)pGpp hydrolase domain (**Figure 1**) (Hogg et al., 2004; Sun et al., 2010; Steinchen and Bange, 2016). This also applies to the hydrolase domain of the bifunctional enzyme Rel_Cg_. An analysis of representatives of all other SAH subgroups showed a very strong conservation of these motifs over all previously identified (pp)pGpp hydrolases (**Figure 1**) (Atkinson et al., 2011). Only the motif HD1 could not be found in the analyzed representatives of the SAH subclasses pbcSpo, pbcSpo2, and divSpo.

### The Deletion of *relH*_Cg_ Causes Only Minor Effects on the Growth Behavior of *C. glutamicum* Under Standard Growth Conditions

In order to investigate the physiological importance of RelH_Cg_, a growth analysis of a *relH*_Cg_ deletion mutant was aimed at. The precise deletion was performed by homologous recombination using the vector pK18*mobsacB*. Since growth effects were observed for mutants with deletions in genes of the (pp)pGpp-synthesizing enzymes Rel_Cg_ and RelS_Cg_ (Ruwe et al., 2017), the gene *relH*_Cg_ was also removed from these single deletion mutants, as well as from the double mutant strain *C. glutamicum* CR099 Δ*rel*, Δr*elS*. CR099 is a direct descendant of the ATCC 13032 wild-type and only the prophage regions (ΔCGP1/2/3) and all members of two families of insertion elements (ISCg1 and ISCg2) have been removed from the chromosome (Baumgart et al., 2013, 2018; Unthan et al., 2015). It is therefore considered as the wild-type (*Cg*) in this study. Shaking flask experiments were performed in CGXII minimal medium and the complex medium CASO broth. To avoid the generation of pseudorevertants in precultures of the growth experiment, as observed for the growth of *E. coli* (p)ppGpp^0^ strains in minimal medium (Murphy and Cashel, 2003), all precultures were grown in complex CASO broth. In the case of the growth experiment in minimal medium, the precultures were subsequently washed in CGXII main culture medium and adjusted to the desired inoculum density.

**FIGURE 2:**
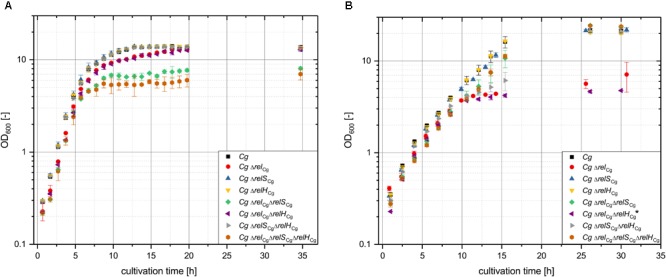
Effect of the deletion of putative (pp)pGpp metabolism associated genes on the growth of *C. glutamicum* CR099 in complex CASO broth **(A)** and minimal CGXII medium **(B)**. To preclude possible enrichment of suppressor mutants all cultures were inoculated from precultures in complex CASO broth. An initial OD_600_ of 0.2 was used for the cultivation in CASO broth. For the growth analysis in CGXII minimal medium, the precultures were washed in the main culture medium and then diluted to an OD_600_ of 0.5. Mean values and standard deviations shown were calculated from three biological replicates. ^∗^In the minimal medium cultivation of the strain CR099 Δ*relS*_Cg_Δ*relH*_Cg_, only two of the three replicas could be evaluated, because the third one showed a deviating growth behavior which did not correspond to the preliminary experiments and was therefore considered to be the result of a pseudoreversion.

For the strain *C. glutamicum* CR099 Δ*relH*_Cg_ no difference compared to the growth behavior of the parental strain was found in both media tested (**Figure 2**). While in minimal medium both strains exhibited exponential growth for the entire cultivation, the use of CASO broth resulted in a course with two characteristic growth phases. Up to an OD_600_ of 4–5 CR099 and CR099 Δ*relH*_Cg_ grew exponentially. Subsequently, after a deceleration phase, a nearly linear growth phase occurred until the final OD_600_ of approximately 13 was reached. This was probably due to a change in the source of nutrients available. As expected, significant differences were observed for all strains in which the long RSH protein encoding gene *rel*_Cg_ was deleted. In complex medium, the corresponding single deletion mutant CR099 Δ*rel*_Cg_ exhibited initially slightly delayed growth and also grew a little bit slower in the second growth phase from an OD of 5-6, compared to the parental strain (**Figure 2A**). In contrast to the unimpaired strains which attained their maximum OD_600_ after 12.5 h, the *rel*_Cg_ deletion mutant reached this value only after 20 h. The additional deletion of *relH*_Cg_ did not affect the growth behavior compared to strain CR099 Δ*rel*_Cg_. An even more pronounced growth deficit was observed for the strain with deletions of both (pp)pGpp synthetase genes, *rel*_Cg_ and *relS*_Cg_. The growth behavior of this strain was similar to that of the Δ*rel* mutant until the second phase. However, after a much stronger deceleration phase, this strain completely stopped growing and reached only an OD_600_ of 8 after 35 h. Again, the additional deletion of *relH*_Cg_ did not result in any further alteration of growth behavior. In summary, no influence of *relH*_Cg_ on the growth behavior could be determined for cultivations in complex medium.

Cultivation in minimal medium basically corresponded to the growth profiles already determined in the analysis of the *C. glutamicum* (pp)pGpp synthetases (Ruwe et al., 2017). Again, the additional deletion of *relH*_Cg_ in the growth affected strains did not lead to further effects. In contrast to the cultivation in complex medium, the strain CR099 Δ*rel*_Cg_Δ*relH*_Cg_ almost completely stopped growing from an OD_600_ of 4, similar to the Δ*rel*_Cg_ single mutant (Ruwe et al., 2017). However, unlike the Δ*rel*_Cg_Δ*relH*_Cg_ double mutant, the strain CR099 Δ*rel*_Cg_ showed an increasing OD value in the cultivation period from 26 to 30 h, following a period of several hours characterized by minimal growth. It should be noted that only two of the three CR099 Δ*rel*_Cg_Δ*relH*_Cg_ replicates were evaluated (**Figure 2B**). The third replicate showed no deceleration phase and exhibited exponential growth over the entire cultivation course, analogous to the parental strain (Supplementary Figure S1). A similar behavior of aberrant replicates has been observed in previous cultivations (data not shown). Since the occurrence of suppressor mutants in RSH deletion mutants is a known phenomenon (Murphy and Cashel, 2003), the two replicates complying with the strain CR099 Δ*rel*_Cg_ were considered correct. According to previous investigations, strains with deletion of both (pp)pGpp-synthetase genes *rel*_Cg_ and *relS*_Cg_ achieved a growth rate corresponding to the parental strain after a significant growth deficit at the beginning of cultivation. Interestingly, when cultivated in CGXII minimal medium, the double deletion mutant CR099 Δ*relS*_Cg_Δ*relH*_Cg_ exhibited a marked growth deficit in the form of an expanded deceleration phase, whereas both single mutants showed no impairment. However, the strain reached a final OD_600_ corresponding to the parental strain after 26 h.

### RelH_Cg_ Exhibits a (pp)pGpp Pyrophosphohydrolase Activity *in Vivo*

In order to test for an *in vivo* activity of the putative (pp)pGpp pyrophosphohydrolase RelH_Cg_, the *relH*_Cg_ gene was heterologously expressed in *E. coli* strains characterized by different (pp)pGpp metabolism variants. In comparison to the *E. coli* wild-type MG1655, strain MG1655 Δ*relA* has a reduced (pp)pGpp basal level (Xiao et al., 1991). Since (pp)pGpp concentration influences the expression of amino acid synthesis genes in *E. coli* and thereby triggers growth effects on suitable minimal medium plates, this combination of strains is well suited to analyze the influence of heterologously expressed (pp)pGpp synthetases and hydrolases (Uzan and Danchin, 1978; Kasai, 2002). In addition to the gene *relH*_Cg_, the already known components of (pp)pGpp metabolism *rel*_Cg_ and *relS*_Cg_ were introduced into the constitutively expressing shuttle vector plasmid pZMP and transformed into the strains *E. coli* MG1655 and *E. coli* MG1655 Δ*relA.*

**FIGURE 3:**
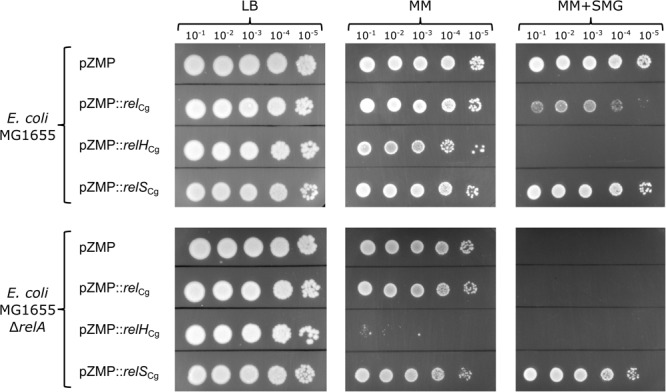
Growth characterization of *E. coli* MG1655 and *E. coli* MG1655 Δ*relA*, each heterologously expressing the *C. glutamicum* genes *rel*_Cg_, *relH*_Cg_ or *relS*_Cg_, on different solid medium plates. The empty vector pZMP was used as control. All strains were cultivated using initial OD_600_ values of 0.05 in LB-medium for 3 h. The cells were washed and serially diluted in PBS-buffer. 5 μL aliquots of every strain and dilution stage were spotted on LB medium plates (LB), minimal medium plates (MM) and minimal medium plates containing each 1 mM L-serine, L-methionine, and L-glycine (MM+SMG). All solid media plates contained 50 μg mL^-1^ kanamycin and 4 g L^-1^ glycerol was used as C source for all minimal medium plates. Incubation was carried out at 37°C for 24 h in the case of LB solid medium and 48 h for MM and MM+SMG plates.

In line with our expectations, no significant growth differences were observed for growth on complex LB medium for all variants of both strains (**Figure 3**). Using minimal medium (MOPS-MM) plates, an almost complete growth inhibition was observed for the expression of *relH*_Cg_ in the strain MG1655 Δ*relA* [(pp)pGpp basal level reduced] compared to the corresponding empty plasmid control. In the wild type strain, however, the expression of the SAH gene only led to minimal reduction in growth. The growth inhibition of the Δ*relA* strain is most likely due to a further reduction of the (pp)pGpp level. Since both strains differ only in the deletion of the monofunctional pyrophosphokinase gene *relA*, no difference in growth behavior would be expected for the wild-type strain if other *relH* associated growth-limiting reasons were responsible for the observed phenotype. Thus, the different effects of *relH*_Cg_ expression in the *E. coli* test strains, in combination with the empty plasmid controls, clearly indicate that RelH_Cg_ exhibits the expected pyrophosphohydrolase activity. Using MOPS-MM plates supplemented with L-serine, L-methionine and L-glycine (SMG-MM) (Kasai, 2002), which induces an even higher (pp)pGpp requirement and thereby inhibits growth of both RelH_Cg_ expressing *E. coli* strains, a significant growth reduction was also triggered by the expression of Rel_Cg_. In analogy to the RelH_Cg_ associated effects, this could be due to a weak constitutive pyrophosphohydrolase activity of Rel_Cg_ *in vivo*. The control strain *E. coli* MG1655 Δ*relA* could only grow under these conditions in the presence of the SAS enzyme RelS_Cg_, whereas the constitutive expression of *rel*_Cg_ had no complementing effect. This is in line with the (pp)pGpp synthesizing activity of RelS_Cg_ (Ruwe et al., 2017).

### RelH_Cg_ Possesses a Mn^2+^ Dependent Pyrophosphohydrolase Activity in Basic pH Range *in Vitro*

In order to analyze the hydrolysis of different alarmone species by RelH_Cg_ and the bifunctional enzyme Rel_Cg_ *in vitro*, both enzymes were expressed heterologously in *E. coli*, purified by affinity chromatography and an *in vitro* pyrophosphohydrolase assay was established. The guanosine derivatives applied as substrate were prepared using the SAS enzyme RelS_Cg_ and purified by precipitation and chromatography steps. Since a strong manganese dependence of the pyrophosphohydrolase reaction was found for the already investigated eukaryotic SAH enzymes as well as bacterial bifunctional RSH enzymes (Aravind and Koonin, 1998; Hogg et al., 2004; Sun et al., 2010), first the dependence of the analyzed enzymes on divalent cations was investigated. In addition, the pH dependence was determined in order to find suitable parameters for further analyses.

**FIGURE 4:**
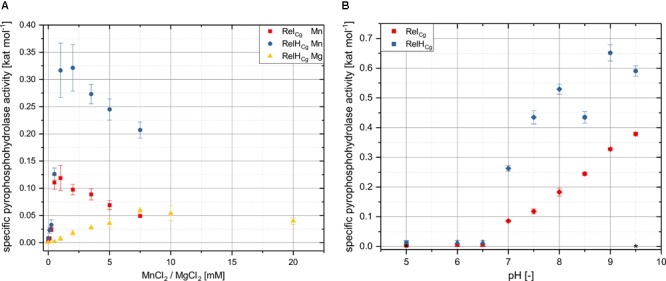
*In vitro* characterization of Rel_Cg_ and RelH_Cg_ with respect to divalent ion concentration and pH. The specific pyrophosphohydrolase activities of the purified enzymes were determined by (pp)pGpp pyrophosphohydrolase assays and subsequent HPLC analysis (see Material and Methods) under variation of MgCl_2_/MnCl_2_ concentration **(A)** and pH-value **(B)**. The reaction mixture for determining the dependence on divalent ions contained 50 mM HEPES-Na (pH 8.0), 200 mM NaCl, 0.5 mM ppGpp as well as 52.3 nM RelH_Cg_ or 98.2 nM Rel_Cg_ respectively, and was incubated at 30°C. In order to avoid oxidation of Mn^2+^, the corresponding solutions were saturated with nitrogen before the addition of substrates and enzyme to remove dissolved oxygen. The incubation time was 25 min for RelH_Cg_ and 45 min for Rel_Cg_ containing approaches. Since comparatively low activities were measured using magnesium, 10 times higher enzyme concentrations were used. The pH dependency was determined with identical reaction components and experimental parameters, whereby different buffer systems and a defined MnCl_2_ concentration of 1 mM were used. The various buffer systems (50 mM each) are illustrated by different icons: square: citrate buffer; triangle: phosphate buffer; rhombus: HEPES-buffer; circle: TRIS buffer; hexagon: ammonia buffer. Mean values and standard deviations shown were calculated from three replicates. ^∗^ Enzyme free controls were carried out at the highest and lowest pH values of 5 and 9.5 and did not yield any unspecific production of GDP or other components, so that the substrate ppGpp is considered to be stable at the pH conditions used.

The suspected Mn^2+^ dependence of the ppGpp hydrolysis was confirmed for both enzymes, as no enzyme activity could be detected without the presence of manganese ions (**Figure 4**). It was found that the maximum activities for the conversion of ppGpp to GDP for Rel_Cg_ and RelH_Cg_ were achieved at manganese concentrations of 1 and 1.75 mM, respectively. Higher Mn^2+^ concentrations led to a significant reduction in pyrophosphohydrolase activity. For RelH_Cg_, the ppGpp hydrolase activity was also detected in the presence of magnesium ions, whereby the maximum activity was achieved at a significantly higher concentration of 7.5 mM. Furthermore, the maximal specific activity of 0.059 kat mol^-1^ was only about 20% of the value of 0.32 kat mol^-1^, which was achieved at 1.75 mM Mn^2+^. For Rel_Cg_ no ppGpp hydrolase activity could be detected in the presence of Mg^2+^ Ions (data not shown). The analysis of pH dependence of both (pp)pGpp hydrolases revealed quite similar characteristics. While both enzymes are inactive in the acid pH range, the specific activity increases continuously with increasing pH from a value of 7. RelH_Cg_ seems to be dependent on the buffer components used, since a remarkably low specific pyrophosphohydrolase activity deviating from the basic trend was measured for a pH value of 8.5 in TRIS buffer.

### The Pyrophosphohydrolase Activities of RelH_Cg_ and Rel_Cg_ Show Substrate Inhibition for Various Alarmone Species

In order to analyze the physiological significance of the two alarmone hydrolases in the context of the (pp)pGpp metabolism of *C. glutamicum*, the enzyme kinetics for the hydrolysis of the three known alarmone species pGpp, ppGpp, and pppGpp were determined. The analysis of the respective activity and specificity parameters for the different substrates is especially interesting to explore the background of different biological functions of the alarmone species (Mechold et al., 2013). In addition, equivalent activity differences were already determined in the course of previous studies of (pp)pGpp synthetases (Steinchen et al., 2015; Ruwe et al., 2017).

**FIGURE 5:**
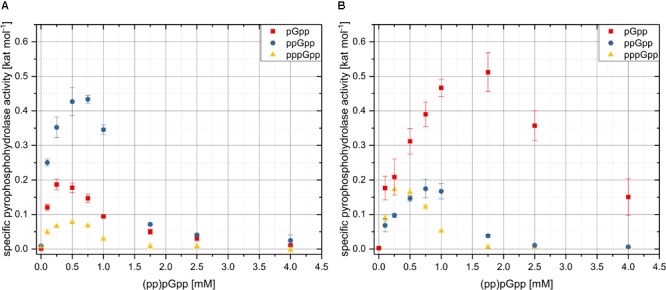
Kinetics of RelH_Cg_ **(A)** and Rel_Cg_ **(B)** using pGpp, ppGpp and pppGpp as substrates. Specific activities were determined for different substrate concentrations by *in vitro* analysis and given in kat per mol of the respective enzyme. The reaction mixtures contained 50 mM HEPES-Na (pH 8.0), 200 mM NaCl, 1 mM MnCl_2_ as well as 52.3 nM RelH_Cg_ or 98.2 nM Rel_Cg_, respectively. The incubation times of the different enzyme-substrate combinations were adapted to the values determined in preliminary tests and ranged from 20 to 45 min. The incubation temperature was 30°C. Mean values and standard deviations shown were calculated from three replicates.

In the course of studying the specific pyrophosphohydrolase activities as a function of the substrate concentration used, fundamentally similar activity levels were found for both Rel_Cg_ and RelH_Cg_. Interestingly, the turnover numbers as well as the corresponding kinetic profiles differed significantly with respect to individual alarmone species (**Figure 5**). Furthermore, a significant reduction in hydrolase activity at high substrate concentrations was observed for both enzymes and all substrates. Therefore, the enzyme parameters *K*_m_ and *k*_cat_ could only be determined by a linearized representation according to Eadie–Hofstee (Hofstee, 1959), using the lowest substrate concentrations (Supplementary Figures S2, S3). For this reason, the values given in **Table 3** are approximate values and would have to be verified using further low substrate concentrations in the uninhibited range of the enzyme kinetics. RelH_Cg_ achieved the highest specific pyrophosphohydrolase activity for the conversion of ppGpp to GDP. The determined turnover number of 0.43 s^-1^ was significantly higher than the values of 0.19 s^-1^ and 0.08 s^-1^ which were calculated for the hydrolysis of pGpp and pppGpp, respectively (**Table 3**). The maximum specific RelH_Cg_ activity was achieved for all substrates already at very low concentrations of 0.25–0.75 mM. Higher concentrations lead to a drastic decrease in activity. At a substrate concentration of 1.75 mM, the specific activities reached only between 10% and 27% of the respective maximum values (**Figure 5**). In contrast, for the hydrolysis activity of the bifunctional enzyme Rel_Cg_, the highest values were obtained using the substrate pGpp. The maximum activity was 0.51 kat mol^-1^ at a pGpp concentration of 1.75 mM. ppGpp and pppGpp were hydrolyzed by Rel_Cg_ with significantly lower maximum hydrolase activities of 0.17 kat mol^-1^ each. In contrast to the kinetics of Rel_Cg_ for pGpp, however, its activity for the hydrolysis of ppGpp and pppGpp collapsed at substrate concentrations of more than 0.75 mM, analogous to the kinetics determined for RelH_Cg_.

**Table 3:** Specific enzyme parameters determined for the hydrolysis of pGpp, ppGpp, and pppGpp by Rel_Cg_ and RelH_Cg_, respectively.

	Rel_Cg_			RelH_Cg_		
Substrate	*K*_m_ [mM]	*k*_cat_ [s^-1^]	*k*_cat_/*K*_m_ [M^-1^ s^-1^]	*K*_m_ [mM]	*k*_cat_ [s^-1^]	*k*_cat_/*K*_m_ [M^-1^ s^-1^]
pGpp	0.734 ± 0.127	0.794 ± 0.071	1.08 × 10^3^	0.144^∗^	0.294^∗^	2.04 × 10^3^
ppGpp	0.511 ± 0.01	0.296 ± 0.004	5.79 × 10^2^	0.101 ± 0.007	0.502 ± 0.018	4.97 × 10^3^
pppGpp	0.422^∗^	0.563^∗^	1.34 × 10^3^	0.09 ± 0.011	0.09 ± 0.003	1.01 × 10^3^

*As a result of significant substrate inhibition, *K*_*m*_ and *k*_*cat*_ were calculated by linearized representation according to Eadie–Hofstee (Hofstee, 1959), using the lowest substrate concentrations (Supplementary Figures S2, S3). ^∗^Due to the substrate inhibition, the parameters shown were only generated on the basis of two data points, which means that no error indication of the regression is possible.*

### The Distribution of the *relH* Homolog in the Genus *Corynebacterium* Indicates a Frequent Gene Loss During Evolution

**FIGURE 6:**
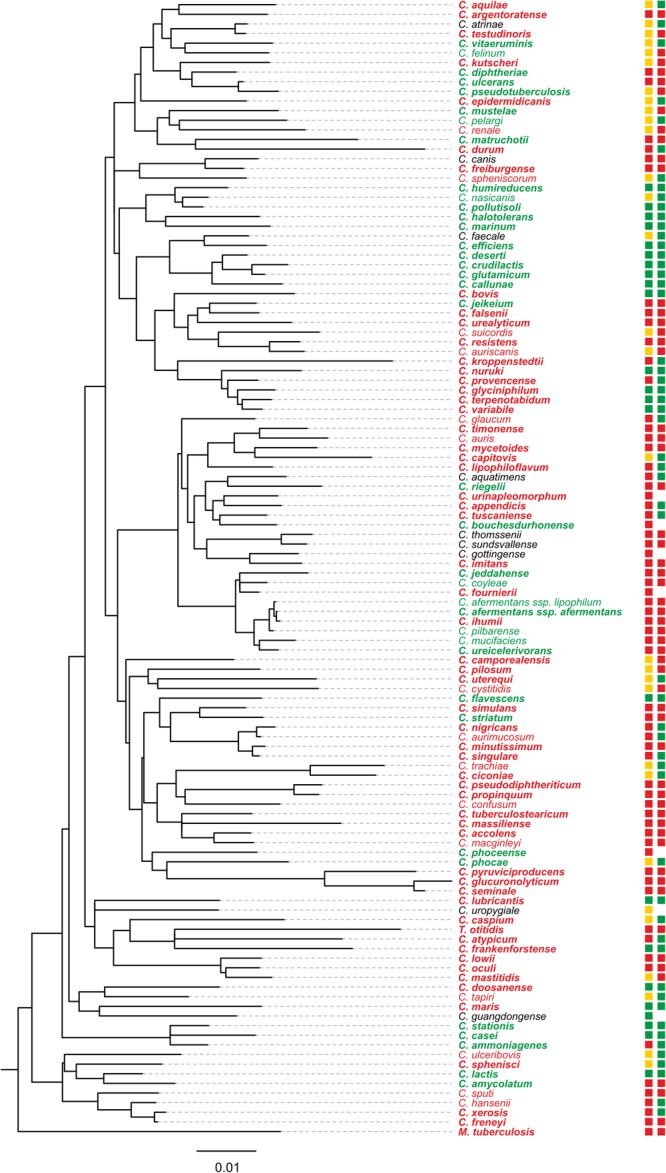
Phylogenetic tree of the genus *Corynebacterium* with additional species-specific information about the occurrence of a *relH*_Cg_ ortholog, the respective isolation site and the biosafety level. The phylogenetic tree was generated on the basis of the respective 16S rDNA sequences using the RDP database (Cole et al., 2014). The analysis of available genomes for the presence of a *relH*_Cg_ ortholog was based on a Hidden Markov model (see Materials and Methods). Species with a respective gene are shown in green, species without *relH* gene in red and species without an available genome in black. The genomes of species printed in bolt type are available in databases, whereas the other genomes are our unpublished data. The first box after the species name represents the isolation site, where green illustrates environmental isolates, yellow those isolated from animals, and red human isolates. The second box corresponds to the biosafety classification (according to German law); green: level one; red: level two; no box: no classification available.

The RelH_Cg_ enzyme appears to constitute an active functional part of the *C. glutamicum* (pp)pGpp metabolism. Since Atkinson et al. (2011) identified representatives of the Mesh-1L subgroup in numerous bacterial groups and in an archaeon during the analysis of the RSH superfamily, studying the *relH*_Cg_ phylogeny represents an interesting approach to generate information on a possible association with specific habitats or lifestyles. An initial BLASTP study revealed that many corynebacteria, in contrast to the highly conserved *rel* and *relS* genes, contain no ORF with a significant similarity to the amino acid sequence of RelH_Cg_ besides the hydrolase domain of the respective *rel* gene. Based on this observation we assessed the presence of this gene in the whole genus of which more than 110 species were described (Oliveira et al., 2017). These species were isolated from the environment but also from humans and animals, where in some cases, a pathogenic potential has been documented. The information obtained was added to a phylogenetic tree based on the 16S rRNA, supplemented additionally with information on the isolation site and the associated biosafety level (**Figure 6**). It was found that species closely related to *C. glutamicum* such as *C. efficiens* and *C. deserti* also possess a *relH* gene. These species represent environmental isolates without known pathogenic association. Interestingly, the more distantly related species of the genus *Corynebacterium* showed a rather heterogeneous occurrence of the analyzed gene. Within the identified phylogenetic subgroups, several representatives lack a *relH* homolog, indicating the occurrence of several independent loss events during evolution. The loss occurs in species isolated from different habitats and both potentially pathogenic and apathogenic species. Since no clear patterns could be observed, there is no apparent association with specific environmental conditions based on the evaluated data. An independent uptake of *relH* orthologs is unlikely due to the high sequence similarities of the existing genes and the resulting delineation within the Mesh-1L subgroup (data not shown).

## Discussion

In this study, the putative SAH gene *relH*_Cg_ (*cg1485*) from *C. glutamicum* was investigated in *in vivo* and *in vitro* analyses with regard to the basic activity of its gene product and its functions within the (pp)pGpp metabolism. Both, the heterologous expression of the *relH*_Cg_ gene in two *E. coli* strains and the hydrolase assay-based study of the purified enzyme confirmed the proposed (pp)pGpp pyrophosphohydrolase activity *in vivo* and *in vitro*. The *in vitro* analysis of the hydrolase activities of RelH_Cg_ and of the bifunctional enzyme Rel_Cg_ with regard to the different alarmone species revealed interesting enzyme characteristics as well as significant differences between both enzymes. The pyrophosphokinase activities of Rel_Cg_ and RelH_Cg_ are strongly dependent on the presence of Mn^2+^ ions. This property fits expectations, as both enzymes exhibit a conserved His-Asp-box motive for Mn^2+^ binding that is characteristic for the HD domain of RSH type hydrolases (Aravind and Koonin, 1998; Mittenhuber, 2001). The corresponding Mn^2+^ dependency of (pp)pGpp hydrolases has already been demonstrated for other organisms (Hogg et al., 2004; Sun et al., 2010). Furthermore, the almost identical pH-dependencies of both (pp)pGpp hydrolases indicate similar reaction mechanisms and comparable enzyme properties. However, while Rel_Cg_ has the highest activity for the substrate pGpp with a comparatively high *K*_m_ value of 0.734 mM, the SAH enzyme RelH_Cg_ prefers the substrate ppGpp with a significantly lower *K*_m_ value of 0.1 mM. The high hydrolysis activity of Rel_Cg_ with respect to pGpp underscores the potential importance of this guanosine derivative which has recently been assigned to the alarmone species (Gaca et al., 2015b). The potential of *C. glutamicum* to synthesize pGpp has already been proven for the SAS enzyme RelS_Cg_ from *C. glutamicum* (Ruwe et al., 2017). The *K*_m_ value of 0.51 mM determined for the hydrolysis of ppGpp by Rel_Cg_ fits well to the data for the Rel ortholog from *Streptococcus equisimilis*, for which a *K*_m_ value of 0.58 mM was determined (Sun et al., 2010). This result illustrates the fundamental comparability of both investigations. In contrast, two analyzed eukaryotic SAH enzymes show significantly higher *K*_m_ values of approximately 3 mM. Furthermore, the maximum activities determined in this study for both Rel_Cg_ and RelH_Cg_ are significantly lower than those identified for eukaryotic SAH enzymes. While RelH_Cg_ possesses a *k*_cat_ value of 0.502 s^-1^ for ppGpp, corresponding measurements for the human SAH enzyme Mesh1 showed a turnover number of 36.6 s^-1^ (Sun et al., 2010). The significant *K*_m_ and *k*_cat_ differences, together with the unknown function of these SAH enzymes in eukaryotic systems, suggest considerable differences. Since no RSH enzyme could be identified so far as (pp)pGpp source for the corresponding organisms, there may even be completely different hyperphosphorylated substrates present. Also, the enzyme Rel_Seq_ from *S. equisimilis* exhibits a more than 10 times higher *k*_cat_ value of 6 s^-1^. In contrast, the catalytic efficiency for ppGpp hydrolysis by Rel_Seq_, with a value of 10.34 × 10^3^ M^-1^ s^-1^ (Sun et al., 2010), is only about twice as high as the efficiency of 4.97 × 10^3^ M^-1^ s^-1^ determined for RelH_Cg_. However, the Rel_Seq_ variant analyzed is a truncated version of the enzyme with an additional base exchange to eliminate its synthetase activity, so that a direct comparability with Rel_Cg_ and RelH_Cg_ is uncertain. Nevertheless, the small turnover numbers of the two (pp)pGpp hydrolases of *C. glutamicum* correspond to the turnover values determined for the (pp)pGpp synthetase activity of the SAS enzyme RelS_Cg_ in a physiological substrate concentration range. At a GDP concentration of 3.125 mM, RelS_Cg_ achieved a specific activity of 0.327 kat mol^-1^ (Ruwe et al., 2017). Therefore, a functional interaction of both monofunctional enzymes in the context of the *C. glutamicum* (pp)pGpp metabolism is realistic on the basis of the determined activity and specificity values.

One of the most interesting observations in this study is the reduction of the pyrophosphohydrolase activity of Rel_Cg_ and RelH_Cg_ at high substrate concentrations. In particular, the activity of RelH_Cg_ decreases significantly at (pp)pGpp concentrations above 0.75 mM. Such substrate inhibition effects may not have been observed so far as insufficient substrate concentrations of maximally 400 μM were used in other studies. The use of these low concentrations is puzzling as the maximum ppGpp concentrations in *E. coli* were found to be in the range of 0.9–4 mM (Sy, 1980; Traxler et al., 2008). However, using classical equations to describe substrate inhibition kinetics based on the formation of an ESS complex, no convergent fit of the experimental data could be obtained when applying realistically dimensioned parameters (Ramsay and Tipton, 2017). The observed behavior therefore is probably not associated with the binding of the substrate to an alternative binding site. Furthermore, the analysis of the inhibition mechanism is complicated by the fact that (pp)pGpp hydrolysis is a reaction with only one substrate. As a result of this, the type of substrate inhibition cannot be verified by varying a non-inhibitory substrate. For the hydrolysis reaction, two further inhibition mechanisms are conceivable. On the one hand, parts of two substrate molecules could bind to the active center of the enzyme and thus form a dead-end ES_2_ complex (Lopina, 2017). The conformational flexibility of the 3′- and 5′-phosphate moieties observed for the alarmone species, which allows interaction with a broad spectrum of intracellular targets (Steinchen and Bange, 2016), could facilitate this process. If an ordered release of the products takes place, which is common for many hydrolytic enzymes in a Ping-Pong type reaction with water as the second substrate, the interaction of the substrate with the enzyme-substrate complex could also take place after the release of the first product (Cleland, 1979). The interaction with this intermediate complex, also known as EQ, in dead-end fashion could also explain the strong decrease in enzyme activity at high substrate concentrations. However, in order to elucidate the molecular background of enzyme behavior, more detailed knowledge of the so far unknown hydrolysis mechanism, in the form of crystal structure analyses or the use of hydrogen deuterium exchange mass spectrometry, is indispensable. During initial analyses of the hydrolysis reaction of long RSH enzymes, for example, the cyclic guanosine intermediate ppG2′:3′p (GPX) was found (Hogg et al., 2004), the function of which has not yet been understood (Steinchen and Bange, 2016). Furthermore, the formation of multimeric enzyme states could make the inhibition mechanism even more complex. A regulatory influence of the oligomerization is already known for long RSH enzymes and SAS representatives (Avarbock et al., 2005; Steinchen et al., 2015; Irving and Corrigan, 2018). The discovery of substrate inhibition in the long RSH representative Rel_Cg_ as well as in the SAH enzyme RelH_Cg_ suggests that this enzymatic property of (pp)pGpp hydrolases may be not limited to *C. glutamicum*. In the context of the stringent response, this effect could be an important part of realizing a bistable system: In response to numerous stressful situations, this global and highly conserved regulatory system activates (pp)pGpp synthetases. The resulting sudden increase in (pp)pGpp levels leads to the activation of a persistence state as the hyperphosphorylated guanosine derivatives influence the biosynthesis or activity of various enzymes. Under these circumstances, the observed substrate inhibition reduces the activity of (pp)pGpp hydrolases which would quickly reduce (pp)pGpp levels back to the basal level otherwise. The substrate inhibition allows the persistence of the alarmone signal while synthesis is turned up, realizing the bistable switch. Indeed, in addition to the genetic network, enzyme properties such as substrate inhibition are known to be elementary components of bistable responses, which allow living cells a specific biochemical response to external stimuli (Tiwari et al., 2011). Moreover, the observed enzyme property may contribute to the realization of specialized (pp)pGpp metabolism-associated functionalities such as phenotypic heterogeneity, which is an important component of the lifestyle of some pathogenic bacteria like *Mycobacterium tuberculosis* (Ghosh et al., 2011). Last but not least, substrate inhibition would avoid an ATP-consuming futile cycle which, given the already limited resources under deficiency conditions, would be detrimental for the cells. Mechanisms that counteract (pp)pGpp futile cycling have already been described for bifunctional long RSH enzymes based on ‘intramolecular regulation of the opposing (p)ppGpp catalytic activities’ (Mechold et al., 2002).

The growth analysis of various RSH mutants in two different media, combined with the results of the *in vitro* analysis of the involved components, provides first insights into the functional relationships of (pp)pGpp metabolism in *C. glutamicum*. For the strains CR099 Δ*rel*_Cg_Δ*relS*_Cg_ and CR099 Δ*rel*_Cg_Δ*relS*_Cg_Δ*relH*_Cg_, growth characteristics similar to that of the parental strain were observed in minimal medium after an initially slightly delayed growth. This is a very interesting result as, according to the present state of knowledge, these strains are (pp)pGpp^0^ strains due to the removal of all (pp)pGpp synthetases, and (pp)pGpp^0^ strains of the model organisms *E. coli* and *Bacillus subtilis* are not capable of growth in minimal medium (Xiao et al., 1991; Kriel et al., 2014). *C. glutamicum* therefore does not seem to require a (pp)pGpp basal level for continuous growth in minimal medium, which illustrates the wide range of (pp)pGpp metabolism associated physiology. A (pp)pGpp^0^ strain which is not auxotrophic for amino acids has already been proven for *Pseudomonas putida* (Bernardo et al., 2009). In contrast to the presumed *C. glutamicum* ppGpp^0^ strains, the single Δ*rel*_Cg_ mutant shows a drastic decline in growth above an OD of 4. This is most likely due to increased (pp)pGpp levels resulting from a pyrophosphokinase activity of RelS_Cg_ in this growth phase combined with the lack of (pp)pGpp hydrolysis activity of Rel_Cg_ (Nanamiya et al., 2008; Ruwe et al., 2017). The additional deletion of RelH_Cg_ causes an additional slightly negative effect on the growth behavior only in the final phase of cultivation from 25 h onward. Therefore, the hydrolase activity of Rel_Cg_ is likely to be the key activity in the degradation of RelS_Cg_ products under these conditions. On the other hand, RelH_Cg_ does not appear to be actively present under these conditions or to participate only insignificantly in the hydrolysis of the RelS_Cg_ products.

The analysis of the available corynebacterial genomes with regard to possible RelH_Cg_ orthologs suggests the function as a complementary (pp)pGpp hydrolase under certain conditions. Numerous species across the genus have apparently lost the corresponding gene in independent events. Therefore, an essential role in the global regulation of cell homeostasis is unlikely. An inadequate hydrolysis of the RelS_Cg_ products by RelH_Cg_ is supported by the unexpected growth deficit of the strain CR099 Δ*relS*_Cg_Δ*relH*_Cg_, whereas both single mutants are not impaired under these conditions. One possible scenario for explaining this issue could be a negative activity coupling of Rel_Cg_ and RelS_Cg_. In the absence of RelS_Cg_, Rel_Cg_ appears to provide the required (pp)pGpp synthesis activity. The negative effect of the additional *relH*_Cg_ deletion suggests that the RelH_Cg_ hydrolase activity represents the counterpart for the synthetically active Rel_Cg_, resulting in a balanced (pp)pGpp level. The lack of RelH_Cg_ would therefore increase the (pp)pGpp levels, analogous to the Δ*rel*_Cg_ situation and thus trigger a growth effect. A study of Rel_Seq_ from *S. equisimilis* supports the previously established hypothesis, as it has shown that long RSH proteins are most likely not active synthetically and hydrolytically at the same time due to a conformation based regulation of their catalytic activities (Hogg et al., 2004).

Synthetase and hydrolase kinetics of the enzymes involved, determined by *in vitro* assays, also support this hypothesis. pGpp, which appears to be produced almost exclusively by RelS_Cg_ (Ruwe et al., 2017), is preferably degraded by Rel_Cg_. This could explain the minimal effects of the additional *relH*_Cg_ gene deletion in the strain CR099 Δ*rel*_Cg_Δ*relH*_Cg_, as pGpp which is produced by RelS_Cg_ may not be sufficiently degraded. However, when interpreting the *in vitro* enzyme characterization, it must be noted that the assays performed represent strongly reduced systems. In particular, the activities of Rel_Cg_ will most likely be significantly influenced by other cellular components. Previous studies have shown that a 12 bp deletion in the large ribosomal subunit protein L11 impairs *C. glutamicum* in (pp)pGpp accumulation upon amino acid starvation (Wehmeier et al., 2001). The amino acid starvation-associated activation of stringent response is therefore probably carried out by a complex of Rel_Cg_, ribosomes and uncharged tRNAs analogous to numerous other organisms (Haseltine and Block, 1973; Avarbock et al., 2000). Furthermore, the activity of the single domain RSH proteins could also be influenced by various cell internal mechanisms. For example, the SAS enzyme RelQ_Bs_ from *B. subtilis* is allosterically activated in its active tetrameric form by its own product pppGpp (Steinchen et al., 2015). This SAS feature also indirectly complies with the substrate inhibition observed for (pp)pGpp hydrolases in this study and may also contribute to the bistable nature of (pp)pGpp metabolism.

In comparison to the cultivation in minimal medium, in which a metabolically quite constant state is present due to the degradation of glucose as the sole carbon source, the growth analysis in amino acid containing complex medium provides further aspects of the (pp)pGpp metabolism in *C. glutamicum*. The cultivation can be divided into two sections. After a period of exponential growth, the wild-type and similarly growing strains enter a second phase with a more linear character. This is most likely due to the consumption of ingredients, which must subsequently be synthesized by *C. glutamicum*. Since the (pp)pGpp^0^ strains have a significant growth deficit in the second cultivation section, the presence of (pp)pGpp seems to be highly relevant under these conditions. Because of the described regulatory effects in other organisms like *E. coli* (Mechold et al., 2013), the alarmone species appear to be crucial for a correct cellular adaptation to new nutritional conditions. For example, an influence of (pp)pGpp on the transcriptional activation of amino acid synthesis clusters due to the consumption of components originally contained in the complex CASO broth is conceivable. Such an influence of the alarmone species on the transcription of amino acid synthesis clusters has already been observed in previous studies as a reaction to the induction of an amino acid starvation in *C. glutamicum* (Brockmann-Gretza and Kalinowski, 2006). The strains CR099 Δ*rel*_Cg_ and CR099 Δ*rel*_Cg_Δ*relH*_Cg_ also exhibit delayed growth in the second cultivation phase, but unlike the presumed (pp)pGpp^0^ strains, they do not completely stop growth and reach the maximum OD with some delay. This can possibly be traced back to increased (pp)pGpp levels due to the lack of Rel_Cg_ hydrolase activity, similar to cultivation in minimal medium. However, insufficient (pp)pGpp levels are also possible, provided that the pyrophosphokinase activities of both Rel_Cg_ and RelS_Cg_ are required under these conditions.

It is important to note that future measurements of the internal (pp)pGpp pools of various mutants are essential to verify the hypotheses based on growth data and *in vitro* enzyme characterization. This is a major challenge as the highly relevant alarmone basal levels are very low and cannot be exactly quantified even with sensitive ^32^P measurements. Using recently established HPLC-based methods for the quantification of messenger nucleotides it is possible to analyze the basal level of ppGpp in *Staphylococcus aureus, Ralstonia eutropha*, and *E. coli* (Kästle et al., 2015; Juengert et al., 2017; Varik et al., 2017). However, the other alarmone species are still below the limit of detection in a non-stress induced state. Moreover, it should be noted that ^32^P-based analyses for *C. glutamicum* have shown that the (pp)pGpp concentrations in this organism are significantly lower than the values determined for *E. coli* (Wehmeier et al., 1998; Tauch et al., 2001). The quantitative analysis of the alarmone species at different growth conditions as well as an gene expression analysis of the functional components could provide important information on the roles of the three known alarmone species, the mechanistic processes during stringent response and the functions of the (pp)pGpp basal level. This could also help to understand the role of (pp)pGpp metabolism in the natural habitat and thus possibly provide valuable information on economically interesting mechanisms such as the growth rate regulation or bacterial persistence and virulence mechanisms.

## Author Contributions

MR, JK, and MP designed, analyzed, and interpreted the performed experiments. JK and MP supervised the research. MR performed the wet lab experiments. CR performed the taxonomic analyses. The manuscript was written by MR and revised by MP, CR, and JK.

## Conflict of Interest Statement

The authors declare that the research was conducted in the absence of any commercial or financial relationships that could be construed as a potential conflict of interest.
