# Supplementary material for: Functional Characterization of a Small Alarmone Hydrolase in Corynebacterium glutamicum
Source: Front Microbiol. 2018 May 9;9:916. doi: 10.3389/fmicb.2018.00916 (PMC5954133; doi:10.3389/fmicb.2018.00916)
Supplement: Supplementary file 1 [file Data_Sheet_1.DOCX]

Supplementary Material

Functional Characterization of a Small Alarmone Hydrolase in *Corynebacterium glutamicum*

**Matthias Ruwe, Christian Rückert, Jörn Kalinowski, Marcus Persicke***

*** Correspondence:** Corresponding Author: [marcusp@cebitec.uni-bielefeld.de](mailto:marcusp@cebitec.uni-bielefeld.de)

**
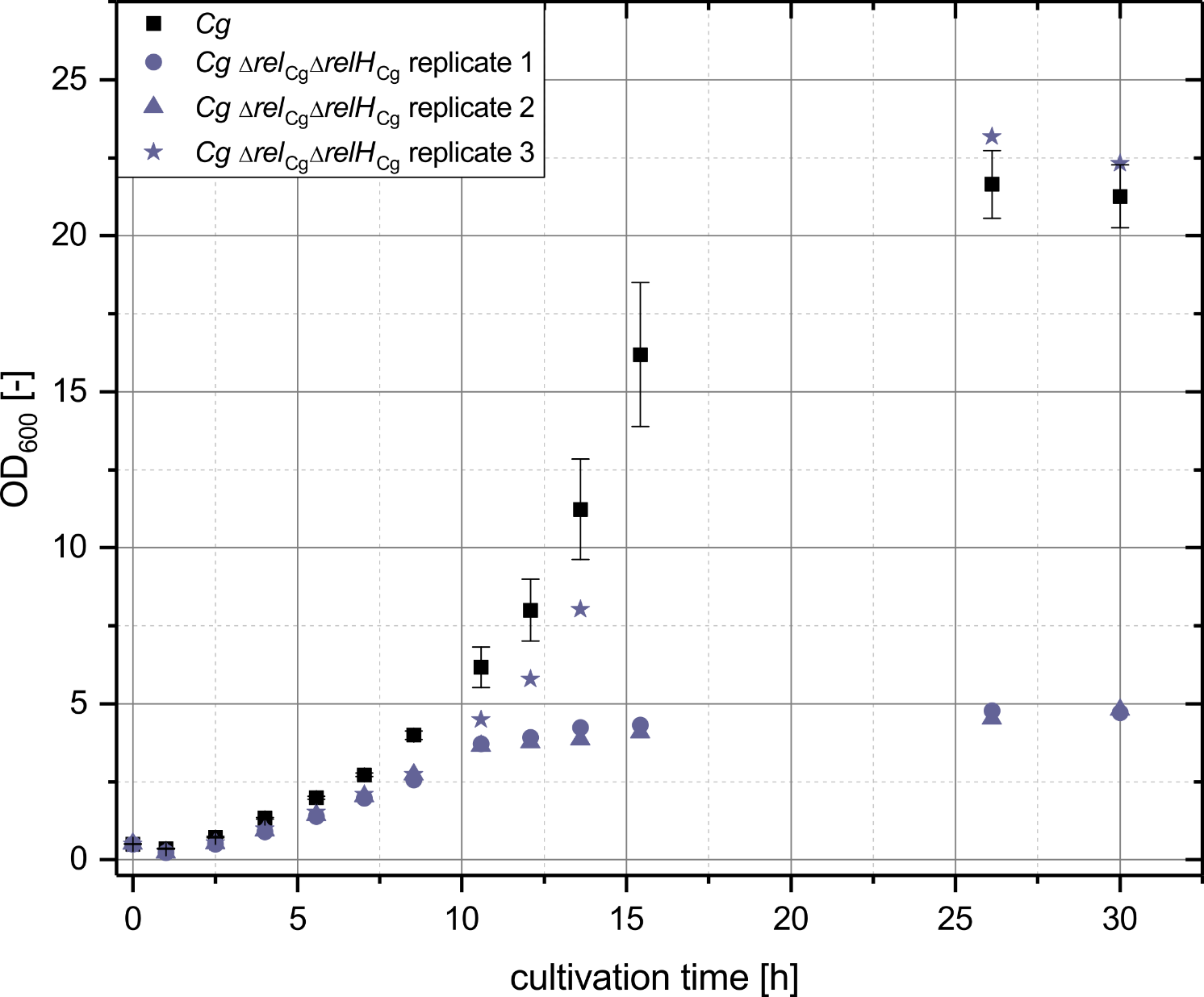
**

**Figure S1.** Growth analysis of three *C. glutamicum* CR099 Δ*rel*_Cg_Δ*relH*_Cg_ replicates, as well as the parental strain in CGXII minimum medium. To preclude possible enrichment of suppressor mutants the cultures were inoculated from precultures in complex CASO broth. The precultures were subsequently washed in the main culture medium and then diluted to an OD_600_ of 0.5. Mean values and standard deviations of the *C. glutamicum* wild type strain were calculated from three biological replicates.


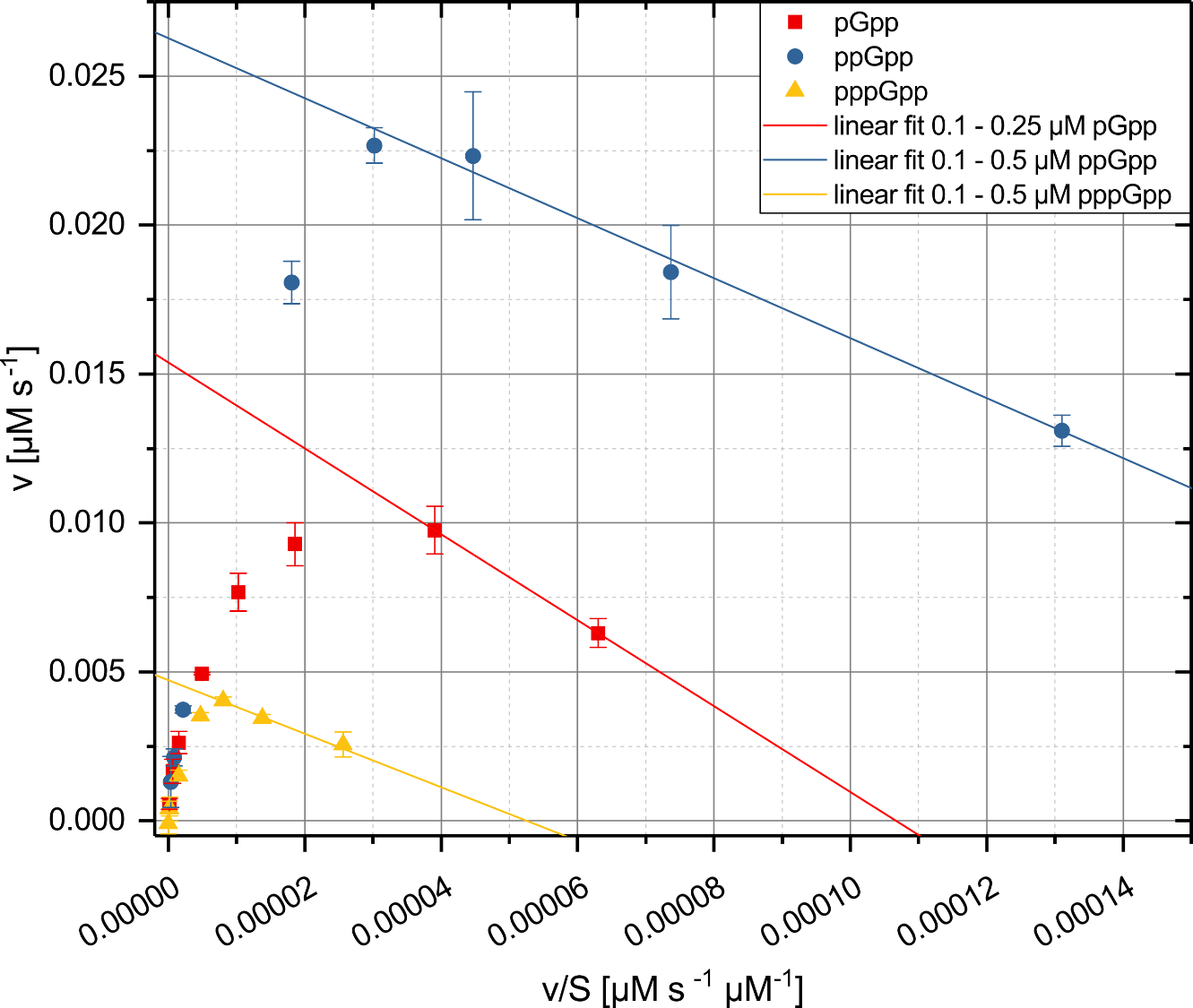


**Figure S2.** Eadie-Hofstee diagram of the RelH_Cg_ kinetics with respect to the concentrations of pGpp, ppGpp and pppGpp as substrates. The reaction rate v [µM s^-1^] was plotted against v/S [µM s^-1^ µM-1]. Due to a considerable substrate inhibition in the substrate concentration range of more than 0.5 mM, the regression used to determine the enzyme parameters k_cat_ and K_m_ was performed only for the linear data range (see diagram legend). For ppGpp only data points could be used, so that no error analysis was possible and the determined parameters merely represent an approximation.


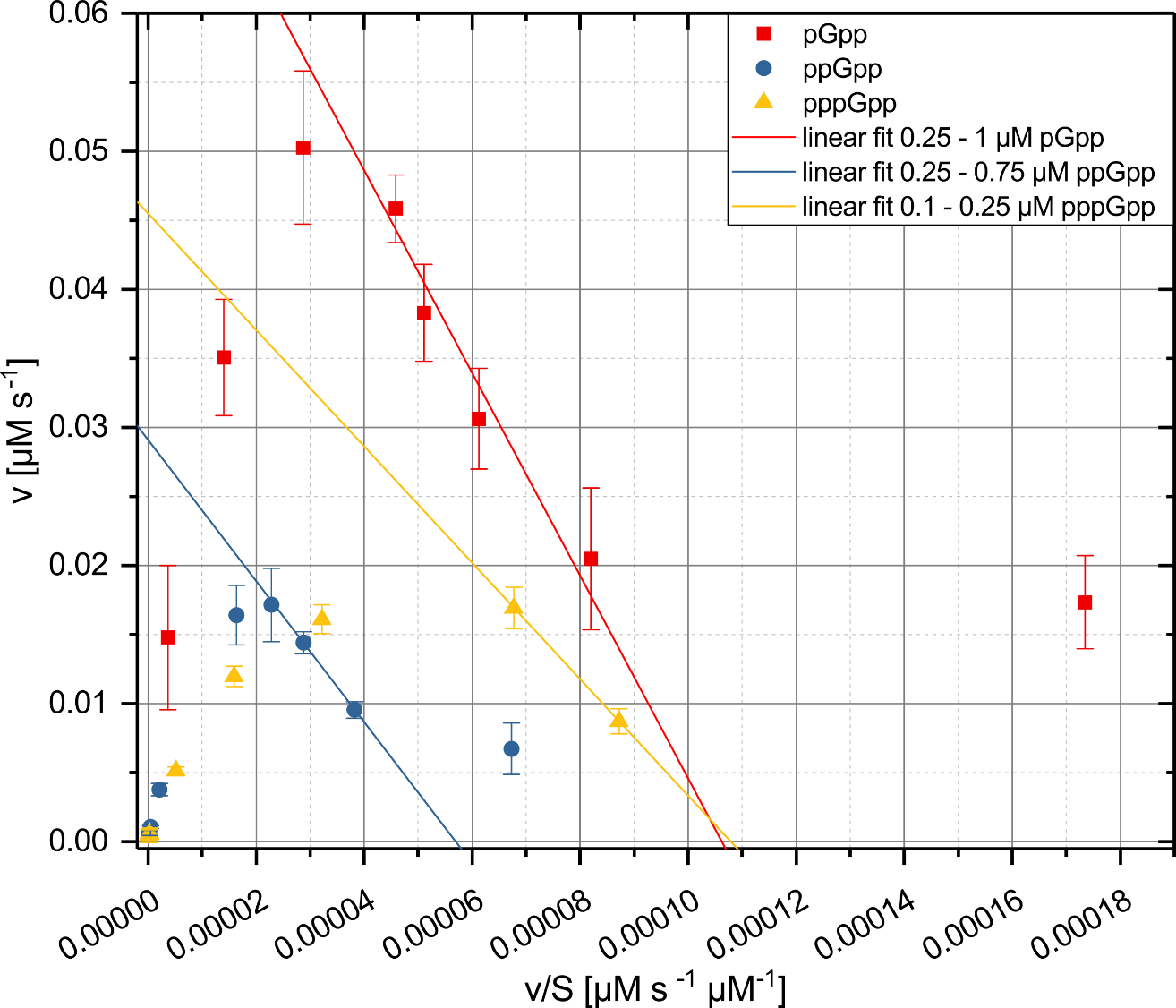


**Figure S3.** Eadie-Hofstee diagram of the Rel_Cg_ kinetics with respect to the concentrations of pGpp, ppGpp and pppGpp as substrates. The reaction rate v [µM s^-1^] was plotted against v/S [µM s^-1^ µM-1]. Due to a considerable substrate inhibition in the substrate concentration range of more than 1 mM, the regression used to determine the enzyme parameters k_cat_ and K_m_ was performed only for the linear data range (see diagram legend). For pppGpp only data points could be used, so that no error analysis was possible and the determined parameters merely represent an approximation.

**Table S1.** Primers used in context of this study. Overlapping sequences, added for Gibson Isothermal Assembly are shown underlined.

| Primer | Sequence | Application |
| --- | --- | --- |
| pZMP_ga1 | GTCTGATAAAACAGAATTTG | Construction of pZMP derivates |
| pZMP_ga2 | GTTTCCTGTGTGAAATTGTT |  |
| pZMP_rel1 | GTGAGCGGATAACAATTTCACACAGGAAACAAAGGAGGACAACATGAGTCTGGAGCGCAACAC | Construction of pZMP::*rel*_Cg_ |
| pZMP_rel2 | TGCCGCCAGGCAAATTCTGTTTTATCAGACCTAGCCACCCGAGGTCACTC |  |
| pZMP_relH1 | GGGCTAGCAGGAGGAATTCACCATGTCAGAGAATTTGCCAGCGCCCGAG | Construction of pZMP::*relH*_Cg_ |
| pZMP_relH2 | CAGCCAAGCTTGCATGCCTGCAGGTTTAAGTGACTTCACTTCCCCGTGG |  |
| pZMP_relS1 | TTTGGGCTAGCAGGAGGAATTCACCATGTCTGACAACACTCTCTC | Construction of pZMP::*relS*_Cg_ |
| pZMP_relS2 | CAGCCAAGCTTGCATGCCTGCAGGTTTAGTTTGGTGGTTCCACAAG |  |
| pK18_ga1 | CTGCAAGGCGATTAAGTTGG | Construction of pK18*mobsacB* derivates |
| pK18_ga2 | CTGGCGTAATAGCGAAGAGG |  |
| pK18_relH1 | TCGGTGCGGGCCTCTTCGCTATTACGCCAGGCACTTACTTTCGCACTCTC | Construction of pK18*mobsacB*_  *relH*_Cg_ |
| pK18_relH2 | CATTGAACCCTAAGCGCTGGCACGCGGCAGTATTCATAGC |  |
| pK18_relH3 | GCTATGAATACTGCCGCGTGCCAGCGCTTAGGGTTCAATG |  |
| pK18_relH4 | CTGGCGTTACCCAACTTAATCGCCTTGCAGGCTGACACCCTCATCGATAC |  |
| pTXB1_ga1 | ATGTATATCTCCTTCTTAAAGTTAAA | Construction of pTXB1 derivates |
| pTXB1_ga2 | TGCATCACGGGAGATGCACTAGTTG |  |
| pTXB1_relH1 | GTTTAACTTTAAGAAGGAGATATACATGTGAATACACTTTCCCCGCGCCTTCG | Construction of pTXB1::*relH*_Cg_ |
| pTXB1_relH2 | CAACTAGTGCATCTCCCGTGATGCAGGCGCTTTGCTTTAAGAGCT |  |
